# Online Psychosocial Intervention for Nursing Students Who Experienced Intimate Partner Abuse in Türkiye

**DOI:** 10.3390/healthcare14080992

**Published:** 2026-04-09

**Authors:** Hacer Demirkol, Şeyda Dülgerler

**Affiliations:** 1 Department of Psychiatric and Mental Health Nursing, Faculty of Health Sciences, Yozgat Bozok University, 66900 Yozgat, Türkiye; hacer.demirkol@yobu.edu.tr; 2 Department of Mental Health and Psychiatric Nursing, Faculty of Nursing, Ege University, Bornova, 35100 İzmir, Türkiye

**Keywords:** intimate partner abuse, posttraumatic stress symptoms, posttraumatic growth, online psychosocial intervention, nursing students

## Abstract

Background/Objectives: Intimate partner abuse (IPA) is common among university students, including nursing students, and is linked to posttraumatic stress symptoms. Accessible online psychosocial interventions are needed to reduce trauma-related symptoms and support posttraumatic growth (PTG). This study examined the effects of an online psychosocial intervention grounded in social learning theory and cognitive behavioral therapy on posttraumatic stress symptoms and PTG among nursing students who experienced IPA in Türkiye. Methods: A randomized controlled trial was conducted among nursing students in Türkiye reporting IPA exposure. Participants were randomly assigned to an intervention group (*n* = 17) or a control group (*n* = 18). The intervention group received an eight-session online psychosocial program delivered individually. Assessments were conducted at pre-intervention, post-intervention, and at 1-, 3-, and 6-month follow-ups. Repeated-measures ANOVA was used, and partial eta-squared (ηp^2^) values were calculated. Results: The intervention group showed significant reductions in posttraumatic stress symptoms compared with the control group, with large effect sizes (*p* < 0.001; ηp^2^ = 0.402–0.676). Furthermore, significant increases were observed in posttraumatic growth, indicating large and sustained effects over time (*p* < 0.001; ηp^2^ = 0.515–0.773). Conclusions: The online psychosocial intervention effectively reduced posttraumatic stress symptoms and enhanced posttraumatic growth among nursing students who experienced IPA. However, results should be interpreted with caution due to the small sample size, and future studies with larger samples are warranted.

## 1. Introduction

Intimate partner abuse (IPA) encompasses a variety of harmful behaviors between romantic and/or sexual partners, including physical, sexual, and psychological mistreatment [1]. IPA represents a pervasive public health concern that affects all levels of society, including university students [2]. Studies conducted worldwide and in Türkiye indicate that the prevalence of IPA among health science students, including nursing students, is alarming, ranging from 15.4% to 53.2% [3,4]. IPA negatively impacts the physical, emotional, and social well-being of university students, ultimately hindering their academic achievement [2]. Among the numerous mental health problems observed after IPA, posttraumatic stress symptoms are particularly prominent [5].

In the literature, posttraumatic stress symptoms are typically evaluated within the diagnostic framework of Post-Traumatic Stress Disorder (PTSD). PTSD is a psychiatric disorder that may develop after individuals experience, witness, or learn about events involving actual or threatened death, serious injury, or sexual violence and is characterized by core symptom clusters including intrusion, avoidance, and hyperarousal. When left unaddressed, posttraumatic stress symptoms may become chronic and develop into PTSD, which is associated with impaired psychological well-being, comorbid mental health conditions such as depression, anxiety, and substance use disorders, and an increased risk of suicide [6]. The prevalence of posttraumatic stress symptoms or PTSD following intimate partner abuse has been reported to reach up to 61% [7].

In addition to negative outcomes, IPA can also be associated with posttraumatic growth (PTG), which involves positive psychological changes and personal strengthening following challenging life events or trauma [8,9]. PTG facilitates improvements in self-perception, interpersonal relationships, and life philosophy. Benefits include enhanced self-awareness, self-confidence, and empathy; stronger personal connections; increased appreciation for life; bolstered faith; and recognition of new opportunities [8,10]. Previous studies indicate that PTG is experienced by victims of intimate partner violence/abuse, including university students [11,12], with the most significant growth observed in areas of spiritual development and exploration of new opportunities [12].

Online interventions have become increasingly common in trauma recovery due to their cost-effectiveness and accessibility. Cognitive behavioral therapy (CBT) is among the most widely used approaches to reduce posttraumatic stress symptoms [13] and promote posttraumatic growth [14]. Although research has identified an association between posttraumatic stress and PTG [15], studies that integrate both outcomes through online interventions remain limited [16,17]. Furthermore, online programs addressing IPA among university students tend to focus on prevention rather than managing trauma-related stress [18]. Consequently, the present study aimed to develop an online psychosocial intervention for nursing students who experienced IPA, addressing both posttraumatic stress symptoms and posttraumatic growth. Based on the literature, the content of the online intervention was informed by principles of social learning theory (SLT) and cognitive behavioral therapy (CBT). Social learning theory emphasizes that behaviors and relational patterns are acquired through observation, modeling, and reinforcement within social contexts. In the context of intimate partner abuse, exposure to gendered norms and relational dynamics, particularly within the family, may contribute to the internalization of victim–perpetrator roles [19,20]. Furthermore, patriarchal social structures and sociocultural norms that position women in subordinate roles and normalize violence within relationships may contribute to the maintenance of abusive dynamics and the perpetuation of violence over time [21]. Although no studies have directly examined the effects of SLT on IPA and PTG, Liu et al. (2020) demonstrated that psychotherapy delivered via videoconferencing, based on social cognitive principles, effectively reduced posttraumatic stress among veterans [22]. Andersson et al. (2021) reported that online CBT interventions for survivors of intimate partner violence significantly reduced posttraumatic stress symptoms [5]. Moreover, multiple studies consistently show that online CBT-based approaches positively affect both posttraumatic stress [13] and posttraumatic growth [16].

Considering the high prevalence of intimate partner abuse among nursing students, there is a need for effective online interventions addressing trauma-related outcomes in this population. Therefore, this study aims to examine the effects of an online psychosocial intervention grounded in social learning theory and cognitive behavioral therapy on posttraumatic stress symptoms and posttraumatic growth among nursing students who have experienced IPA in Türkiye.

The hypotheses of the study are as follows:

**H_1_:**  *The online psychosocial intervention will effectively reduce the severity of posttraumatic stress symptoms among nursing students who have experienced IPA.*

**H_2_:**  *The online psychosocial intervention will effectively enhance posttraumatic growth levels among nursing students who have experienced IPA.*

## 2. Materials and Methods

### 2.1. Design

A randomized controlled trial was conducted with five repeated assessments. The study included the following assessments: pretest (pre-intervention), posttest (immediately after completing the intervention), 1st-month follow-up (one month after the intervention), 3rd-month follow-up (three months after the intervention), and 6th-month follow-up (six months after the intervention). The study followed the CONSORT E-HEALTH statement [23]. Trial registration: ClinicalTrials.gov identifier: NCT05368779 https://clinicaltrials.gov/ct2/show/NCT05368779 (accessed on 10 May 2022).

### 2.2. Participants and Sampling

A total of 371 undergraduate nursing students from a health sciences faculty in Türkiye were screened for eligibility to participate in the online psychosocial intervention. The study was conducted at a public university where the researcher is affiliated; therefore, sampling was based on accessibility and feasibility rather than random institutional selection. The assessment included the Introductory Information Form (IIF), Suicide Probability Scale (SPS), Brief Symptom Inventory (BSI), Somatoform Dissociation Questionnaire (SDQ), and the Impact of Event Scale-Revised (IES-R) and was conducted through an initial screening followed by a more detailed eligibility assessment.

Inclusion criteria were: (i) being at least 18 years old; (ii) having access to a computer and the internet; (iii) being able to read and understand Turkish; (iv) experiencing intimate partner abuse (self-reported); (v) having ended the relationship with the abusive partner, with at least one month elapsed since the breakup; (vi) being on a stable psychiatric medication dose for at least the last three months or medication-free; and (vii) scoring 24 or higher on the IES-R, the cutoff for probable PTSD in Turkish validation studies [24]. Selecting participants who had ended their relationships with abusive partners was particularly important due to the high rate of femicide in Türkiye.

Exclusion criteria included: (i) being married, as the study specifically focused on intimate partner abuse in non-marital (dating) relationships among university students, where such relationships are more common and differ from marital relationships in terms of relational dynamics, commitment, and help-seeking patterns, in order to ensure a more homogeneous sample; (ii) having a high suicide risk (SPS ≥ 110); (iii) showing psychotic symptoms (BSI psychoticism subscale > 2); (iv) exhibiting high dissociative symptoms (SDQ ≥ 35), corresponding to clinically significant levels of dissociation. These individuals were excluded due to safety considerations, as they may be at risk of psychological destabilization during trauma-related content, in line with previous internet-based interventions [25]; (v) engaging in risky alcohol or substance use; and (vi) receiving non-drug psychiatric treatment (e.g., CBT, psychoanalytic therapy) [26,27]. Students with psychiatric symptoms requiring intervention or who were identified as at risk of suicide were referred to appropriate medical care.

**Figure 1:**
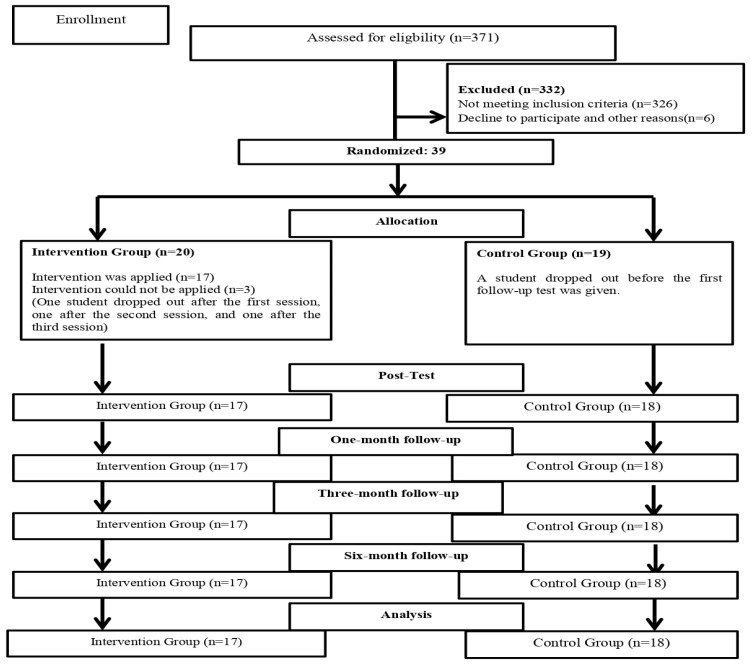
CONSORT-E HEALTH flowchart of study participants, point of random assignment, and dropouts at each stage.

Of the screened students, 39 met the inclusion criteria and volunteered to participate. Participants were classified by sex and social support, as these variables may influence posttraumatic stress and growth [8,28], and randomization was conducted by an independent researcher using an online random number generator [29]. After randomization, four participants were lost to follow-up. In the intervention group, three participants did not complete the study: two withdrew due to COVID-19 infection, and one withdrew after initiating antidepressant and anxiolytic treatment. In the control group, one participant withdrew due to COVID-19 infection. Therefore, the randomized controlled trial was completed with 35 participants (intervention, *n* = 17; control, *n* = 18). Details on sampling and attrition are presented in Figure 1.

Based on meta-analytic evidence indicating that internet-delivered cognitive behavioral therapy (iCBT) for posttraumatic stress disorder yields medium to large effect sizes (g = 0.71, 95% CI [0.49–0.93]) [30], a power analysis conducted using G*Power version 3.1.9.4 indicated that a minimum total sample size of 34 participants would be required (α = 0.05, power = 0.80). In the present study, 39 nursing students were recruited, and the study was completed with 35 participants (intervention = 17; control = 18). Based on the final sample size, a post hoc power analysis demonstrated a statistical power of 0.99 for posttraumatic stress symptoms as measured by the IES-R.

### 2.3. Measures

#### 2.3.1. Initial Measurements

##### Introductory Information Form (IIF)

The Introductory Information Form (IIF) was developed by the researchers based on relevant literature [8,26,27,28] to collect both initial screening and detailed sociodemographic and clinical information and consisted of 20 items. The form included items assessing age, gender, perceived social support, exposure to intimate partner abuse (IPA), relationship status with the abusive partner, frequency and amount of alcohol use, substance use, non-drug psychiatric treatment, and the use and duration of psychiatric medication. Items were structured as dichotomous (yes/no) and categorical questions.

Exposure to IPA was assessed through self-report using dichotomous (yes/no) items indicating the presence of specific types of abuse, including physical, emotional, sexual, and economic abuse. This assessment captured the presence and type of abuse but did not include a detailed evaluation of frequency or severity. Break-up with the abusive partner was operationalized as a dichotomous (yes/no) variable indicating whether the participant had ended the relationship with the abusive partner. Participants were classified as having ended the relationship if they reported that they were no longer in an ongoing relationship with the abusive partner and that at least one month had elapsed since the breakup at the time of data collection.

##### Suicide Probability Scale (SPS)

Cull and Gill (1990) [31] developed it as a self-report scale to assess the risk of suicide in adults. It is a four-point Likert scale with 36 items. The total score obtained from the scale ranges between 36 and 144, with high scores indicating a high risk of suicide [31]. The internal consistency of the SPS was reported as 0.89 in the Turkish validity and reliability study. The cutoff point of the scale for Turkish people in terms of suicide risk was determined to be 110 [32].

##### Brief Symptom Inventory (BSI)

The BSI, derived from the Symptom Checklist-90-Revised (SCL-90-R), was developed by Derogatis (1992) [33] and consists of 53 items and nine subscales. The subscales include somatization, obsessive-compulsion, interpersonal sensitivity, depression, anxiety, hostility, phobic anxiety, paranoid ideation, and psychoticism [33]. In the Turkish validity and reliability study, Sahin and Durak (1994) reported that the Cronbach’s alpha coefficients for the subscales varied across different samples and analyses, ranging from approximately 0.55 to 0.86 [34]. Only the psychoticism subscale was used in this study. To ensure participant safety and the appropriateness of the intervention, individuals with elevated symptom levels (mean score > 2), corresponding to at least moderate symptom severity, were excluded based on an operational threshold. Excluding individuals with elevated psychotic symptoms is consistent with prior trauma-focused and internet-based intervention studies [25,27].

##### Somatoform Dissociation Questionnaire (SDQ)

The SDQ, developed by Nijenhuis et al. (1996), assesses somatoform dissociation symptoms and consists of 20 items [35]. The Turkish validity and reliability of the scale were examined by Sar et al. (2001) [36], who reported a Cronbach’s α coefficient of 0.94. The cutoff score for the Turkish population was determined to be 35 [36].

##### Impact of Event Scale–Revised (IES-R)

The Impact of Event Scale–Revised (IES-R), based on the original scale developed by Horowitz et al. (1979) [37], was revised by Weiss and Marmar (1997) to assess posttraumatic stress symptoms over the past seven days [38]. The Turkish validity and reliability of the scale were examined by Corapcıoglu et al. (2006) [24], who reported an internal consistency coefficient of 0.937. The scale consists of 22 items and includes three subscales: intrusion, avoidance, and hyperarousal. The Turkish version of the IES-R has demonstrated good diagnostic performance, with suggested cutoff values ranging from 24 to 33 for posttraumatic stress symptoms [24]. The IES-R is one of the most widely used self-report instruments for assessing PTSD symptoms worldwide. However, it is not a diagnostic tool and is primarily used for screening purposes; therefore, a clinical assessment is required for a definitive PTSD diagnosis [39].

#### 2.3.2. Outcome Measurements

The IES-R was used in this study to assess posttraumatic stress symptoms, and the Posttraumatic Growth Inventory (PTGI) was used to assess posttraumatic growth. The outcomes were measured in both the intervention and control groups before and after the intervention, and at the 1-, 3-, and 6-month follow-ups.

##### Posttraumatic Growth Inventory (PTGI)

The PTGI, comprising a total of 21 items, was developed by Tedeschi and Calhoun (1996) [40] to assess posttraumatic growth. The scores range from 0 to 105, with higher scores indicating greater perceived posttraumatic growth resulting from traumatic experiences. The PTGI has acceptable internal consistency for both the total scale (α = 0.90) and subscales (Relating to others: α = 0.85; New possibilities: α = 0.84; Personal strength: α = 0.72; Spiritual change: α = 0.85; Appreciation of life: α = 0.67) [40]. Kagan et al. (2012) [41] investigated the validity and reliability of the PTGI in Turkish university students. Unlike the original scale, the Turkish version of the PTGI is divided into three subscales: “Change in Self-Perception,” “Change in Philosophy of Life,” and “Change in Relationships,” which collectively cover the five subscales of the original scale. In the Turkish validation study, the Cronbach’s α coefficient was reported as 0.92 [41]. Consistent with prior research, the cutoff score for this study was set at 63 [42,43].

### 2.4. The Online Psychosocial Intervention

The content of the online psychosocial intervention was developed based on social learning theory (SLT) and cognitive behavioral therapy (CBT) [8,20,28,44]. Prior to implementation, a pilot study was conducted with four students; data obtained from the pilot study were not included in the main analyses.

The online psychosocial intervention was delivered to the intervention group via Zoom videoconferencing over eight weekly sessions, each lasting 90–120 min. Homework assignments from each session were reviewed at the beginning of the subsequent session. The intervention was delivered by H.D., who holds a doctorate in psychiatric nursing and is a certified CBT therapist accredited by the European Association for Behavioral and Cognitive Therapies. Sessions were supervised once per week by the second researcher, Ş.D., who is also a certified CBT therapist.

All intervention sessions were videotaped to allow participants to review the recordings independently as part of the intervention process. Nursing students in the control group were contacted once per week via text message to assess their general mental health status, and no additional intervention was provided. After completion of the follow-up assessments, the online psychosocial intervention was offered to three volunteer students in the control group.

All participants were instructed to contact the research team in the event of suicidal ideation. Throughout both the intervention period and the follow-up assessments, participants were asked whether they experienced any changes in psychiatric medication or received additional psychiatric treatment. An experienced CBT therapist (H.D.) was available for emergencies, particularly in cases of suicidal risk; however, no emergency intervention was required. Additionally, during the follow-up period, participants were asked whether they had experienced any traumatic events other than intimate partner abuse, and all participants reported that they had not. The content of the online psychosocial intervention is presented in Table 1.

### 2.5. Data Analysis

Statistical analyses were conducted using IBM SPSS Statistics for Windows (IBM Corp., Armonk, NY, USA), version 25.0. All tests were evaluated at a 95% confidence level with a significance level of *p* < 0.05. Demographic data were analyzed using descriptive statistics. Chi-square analyses were conducted to compare the intervention and control groups regarding sex, social support, and type of intimate partner abuse. Independent-samples *t*-tests were conducted to examine baseline differences between the intervention and control groups in terms of IES-R and PTGI total scores. Repeated-measures analysis of variance (ANOVA) was used to examine differences over time between the intervention and control groups for IES-R and PTGI total and subscale scores. Effect sizes for group × time interactions were evaluated using partial eta-squared (ηp^2^). Effect sizes were interpreted based on commonly used benchmarks, where values of 0.01, 0.06, and 0.14 indicate small, medium, and large effects, respectively [45]. Given the minimal attrition, analyses were conducted using a complete-case approach, which was not expected to bias the results.

## 3. Results

### 3.1. Data on the Sociodemographic and IPA-Related Characteristics of the Nursing Students

The mean age of the participants was 21.45 ± 1.63 years (range: 18–25). The majority of participants were women (*n* = 26), and all identified as Muslim. Chi-square analyses revealed no statistically significant differences between the intervention and control groups with respect to sex or adequate social support, which were used as randomization criteria. Additionally, no significant differences were observed between the groups regarding the types of intimate partner abuse experienced (*p* > 0.05) (Table 2).

### 3.2. Data on Posttraumatic Stress Symptom Scores and Their Effect Sizes

Before the online psychosocial intervention, students in both the intervention (M = 45.94, SD = 14.41) and control groups (M = 51.61, SD = 10.97) had IES-R total mean scores that were above the cutoff score of 24, with no statistically significant difference between the groups at baseline (t = −1.314, *p* > 0.05). Following the intervention, the IES-R total mean score of the intervention group decreased below the cutoff score of 24 and remained below this threshold throughout the six-month follow-up period. In contrast, the IES-R total mean score of the control group approached the cutoff level by the six-month follow-up (Table 3).

Repeated-measures analysis of variance revealed statistically significant group × time interactions for the IES-R total score (F = 14.901, *p* < 0.001) as well as for the intrusion (F = 9.445, *p* < 0.001), avoidance (F = 8.660, *p* < 0.001), and hyperarousal (F = 10.611, *p* < 0.001) subscales. The effect sizes for the group × time interactions were large [45], with partial eta-squared values of ηp^2^ = 0.622 for intrusion, ηp^2^ = 0.402 for avoidance, ηp^2^ = 0.636 for hyperarousal, and ηp^2^ = 0.676 for the IES-R total score (Table 4).

### 3.3. Data on Posttraumatic Growth (PTG) Scores and Their Effect Sizes

Before the online psychosocial intervention, the PTGI total mean scores of students in the intervention (M = 33.05, SD = 5.35) and control groups (M = 29.44, SD = 6.84) were well below the cutoff score of 63, with no statistically significant difference between the groups at baseline (t = 1.733, *p* > 0.05). Following the intervention, the PTGI total mean score of the intervention group increased above the cutoff score and remained above this threshold throughout the six-month follow-up period, whereas the control group did not reach the cutoff score at any assessment point (Table 3).

Repeated-measures analysis of variance revealed statistically significant group × time interactions for the PTGI total score (F = 150.801, *p* < 0.001) as well as for the subscales of change in self-perception (F = 136.877, *p* < 0.001), change in life philosophy (F = 44.170, *p* < 0.001), and change in relationships (F = 79.879, *p* < 0.001). The effect sizes for the group × time interactions were large [45], with partial eta-squared values of ηp^2^ = 0.762 for change in self-perception, ηp^2^ = 0.515 for change in life philosophy, ηp^2^ = 0.605 for change in relationships, and ηp^2^ = 0.773 for the PTGI total score (Table 4).

## 4. Discussion

The purpose of this study was to examine the effectiveness of an online psychosocial intervention grounded in SLT and CBT on posttraumatic stress symptoms and posttraumatic growth among student nurses who had experienced IPA.

Although IPA is quite common among university students, including nursing students [2,3,4], only a limited proportion of students disclosed exposure and agreed to participate in the online psychosocial intervention. Interestingly, despite evidence in the literature suggesting that men also experience IPA [46], the number of male students reporting exposure and agreeing to participate in the intervention was very limited. A study conducted in Türkiye by Ozturk et al. (2021) among health-related students, including nursing students, reported a comparable prevalence, with 15.4% of participants indicating exposure to dating violence in their current relationships [3].

The study was conducted in a region of Türkiye characterized by prevalent patriarchal and conservative social structures, which may help explain the low reporting of IPA among nursing students. Although the study setting was selected based on accessibility and feasibility rather than specific sociocultural criteria, these contextual characteristics should be considered when interpreting the findings. In such contexts, patriarchal and conservative sociocultural norms may contribute to keeping intimate partner abuse within the private sphere and inhibiting its disclosure. These norms may also contribute to the normalization of abusive behaviors, as described in the normalization of violence perspective [47], whereby such behaviors may not be perceived as problematic or may remain unrecognized [21]. Additionally, cultural and religious norms emphasizing privacy, honor, and traditional gender roles may further reinforce these patterns and discourage help-seeking behaviors [20,44]. Moreover, within such a patriarchal context, a male student’s disclosure of IPA may be perceived as a challenge to his social power and may expose him to stigma or homophobic reactions [46]. Given these factors, it is essential to thoroughly investigate IPA and to clarify students’ perspectives through qualitative research methods, particularly considering its adverse effects on students’ physical and mental health and educational outcomes in conservative and patriarchal societies such as Türkiye [20,46,48].

Emotional abuse often co-occurs with other forms of intimate partner abuse. The findings of the present study suggest that nursing students were able to recognize their experiences of emotional abuse, despite the fact that this form of abuse is often difficult to identify and acknowledge [49]. This awareness may be partly attributable to the inclusion of social issues such as intimate partner abuse and gender equality in the nursing curriculum at the faculty where the study was conducted. Furthermore, the integration of IPA and related topics into nursing curricula globally, particularly in patriarchal contexts, may further strengthen students’ capacity to recognize their own abusive experiences and to understand and support individuals exposed to abuse [20,21,46,48].

According to the findings of the present study, the online psychosocial intervention developed using SLT and CBT and implemented via the videoconferencing method is a long-term effective intervention to reduce posttraumatic stress symptoms after IPA. Similar studies conducted with individuals with PTSD, including individuals who were IPA survivors, have shown that online interventions based on social and cognitive models are effective in reducing posttraumatic stress levels in the long term [5,22,26].

In this study, the control group exhibited reductions in posttraumatic stress symptoms at both the three- and six-month follow-ups. These findings are consistent with the literature, as some studies similarly report decreases in posttraumatic stress symptoms among control groups that did not receive any intervention [25,50]. Moreover, in a small subset of individuals, posttraumatic stress symptoms may remain elevated for three to six months and potentially become chronic [13,28].

The online psychosocial intervention implemented in this study demonstrated substantial effectiveness in reducing posttraumatic stress symptoms among university students who had experienced intimate partner abuse (IPA). Large effect sizes were observed across the IES-R subscales and total scores (ηp^2^ = 0.402–0.676) [45]. These results are in line with previous findings by Andersson et al. (2021), who reported a large between-group effect size (d = 0.89) for the IES-R following an online CBT program for survivors of intimate partner violence [5]. Despite differences in cultural and contextual factors between the studies, both provide converging evidence that online interventions grounded in social-cognitive principles can yield significant reductions in posttraumatic stress symptoms. Nonetheless, caution is warranted in interpreting these effect sizes, given the relatively small sample size in the present study. Future research with larger and more diverse samples is needed to replicate and confirm the magnitude of these effects.

The findings of this study indicate that online psychosocial interventions exert a sustained positive effect on posttraumatic growth (PTG) among university students who have experienced intimate partner abuse (IPA). While no previous research has specifically examined the effects of online interventions grounded in social learning theory (SLT) on PTG, prior studies have demonstrated that CBT-based interventions can facilitate posttraumatic growth [16,17]. To our knowledge, this study is the first to investigate the combined effect of SLT and CBT delivered via an online format on PTG in this population. Large effect sizes were observed for the PTGI total score, both in terms of between-group differences and group × time interactions [45], suggesting that online psychosocial interventions may represent an effective approach to enhance PTG. In contrast, no posttraumatic growth was observed in the control group over the six-month follow-up period. Nevertheless, previous studies indicate that some university students may experience PTG even in the absence of intervention [11,51]. These findings highlight the importance of designing online interventions that account for factors known to influence posttraumatic growth, including the nature of the trauma, cultural characteristics (e.g., spirituality and social support), and individual differences [8].

The present study has several limitations that should be acknowledged when interpreting the findings. First, the relatively small sample size may restrict the generalizability of the results, and the reported effect sizes should therefore be interpreted with caution. Replication in larger and more diverse samples is necessary to confirm the robustness and reliability of these findings. Second, posttraumatic stress symptoms were assessed using the IES-R, a self-report instrument, rather than structured clinical interviews, which may have introduced reporting bias. Future studies would benefit from incorporating clinician-administered diagnostic assessments to enhance the validity and precision of outcome evaluation. Third, trauma exposure was operationalized solely as intimate partner abuse, although participants may have experienced additional traumatic events that could have influenced both posttraumatic stress symptoms and posttraumatic growth. Future research should consider assessing cumulative or multiple trauma exposures to provide a more comprehensive understanding of intervention effects and to more accurately evaluate the efficacy of psychosocial interventions. Additionally, the exclusion criteria applied in this study, such as marital status and dissociative symptoms, may limit the generalizability of the findings; therefore, the results should be interpreted with caution.

## 5. Conclusions

The online psychosocial intervention, grounded in social learning theory and cognitive behavioral therapy and delivered across eight sessions, was effective in reducing posttraumatic stress symptoms among nursing students who had experienced IPA. In addition to alleviating posttraumatic stress symptoms, the intervention also promoted posttraumatic growth, with benefits maintained over the six-month follow-up period. However, given the relatively small sample size, the observed effect sizes should be interpreted with caution, and replication in studies with larger samples is warranted. Future research should tailor online psychosocial interventions to the needs and cultural contexts of university students experiencing IPA and include comparisons between face-to-face and online delivery formats using randomized controlled designs.

## Figures and Tables

**Table 1:** The online psychosocial intervention content.

Sessions	Content
First Session	Psychoeducation about intimate partner abuse (IPA) and its prevalence as a common experience (normalization). Psychoeducation about trauma and trauma-related symptoms, with an emphasis on increasing awareness of individual trauma symptoms and evaluation of sociocultural factors. Introduction and guided practice of relaxation techniques, including deep breathing, progressive muscle relaxation, and safe place imagery.
Second Session	Psychoeducation on cognitive behavioral approaches and exposure techniques. Application of imaginal exposure to partner abuse experiences. Distress levels were intermittently assessed using a Visual Analog Scale (VAS), and relaxation techniques were applied when necessary during the session. *Homework:* Independent monitoring of the second session recording and completion of the Emotion–Thought–Behavior Chart (a structured tool used to identify and link individuals’ emotions, thoughts, and behaviors in response to specific situations).
Third Session	Discussion of experiences arising from monitoring of the second session recording. Continued imaginal exposure to partner abuse experiences. Continued examination of emotion–thought–behavior connections, automatic thoughts, cognitive distortions, intermediate beliefs, and core beliefs related to past partner abuse experiences (cognitive reframing). *Homework:* Independent monitoring of the third session recording and completion of the Emotion–Thought–Behavior Chart.
Fourth Session	Continued discussion of experiences arising from monitoring of the third session recording. Continued imaginal exposure to process partner abuse experiences. Continued cognitive reframing focusing on emotion–thought–behavior connections, automatic thoughts, cognitive distortions, intermediate beliefs, and core beliefs related to past partner abuse experiences (cognitive reframing). *Homework:* Independent monitoring of the fourth session recording and completion of the Emotion–Thought–Behavior Chart.
Fifth Session	Continued discussion of experiences arising from monitoring of the fourth session recording. Addressing partner abuse experiences within the present context, focusing on problem-focused solution strategies (e.g., identifying practical steps to manage interpersonal difficulties or seeking social support) and discussion of alternative thoughts. *Homework:* Incorporation of problem-focused solution techniques into daily life and completion of the Emotion–Thought–Behavior Chart.
Sixth Session	Application of problem-focused solution techniques in real life. Addressing partner abuse experiences within the present context, focusing on problem-focused solution strategies and discussion of alternative thoughts. Discussion of psychological pain and posttraumatic growth. Acceptance of the universality of pain and integration of partner abuse experiences into life history. *Homework:* Writing a narrative acknowledging partner abuse, listing personal values and strengths, and completion of the I, My Values, and My Future Chart.
Seventh Session	Discussion of participants’ narratives, acknowledging their experiences of partner abuse. Discussion of participants’ strengths, values, future goals, and perspectives on a meaningful life. *Homework:* Writing value-based objectives and a letter to a future romantic partner.
Eighth Session	Continued discussion of participants’ strengths, values, future goals, and perspectives on a meaningful life. Discussion of letters written to future partners. Comprehensive review and evaluation of the entire intervention process.

**Table 2:** Comparison of sociodemographic and IPA-related characteristics between the intervention and control groups.

Characteristics		Intervention Group		Control Group		χ^2^ *p*
		**n**	**%**	**n**	**%**	
Gender	Female	13	76.5	13	72.2	0.083, 0.540
	Male	4	23.5	5	27.8	
Adequate social support	Yes	8	47.1	8	44.4	0.024, 0.573
	No	9	52.9	10	55.6	
Physical abuse victimization	Yes	11	64.7	12	66.7	0.015, 0.592
	No	6	35.3	6	33.3	
Emotional abuse victimization	Yes	17	100.0	18	100.0	-
	No	0	0.0	0	0.0	
Sexual Abuse victimization	Yes	3	17.6	4	22.2	0.114, 0.534
	No	14	82.4	14	77.8	
Economic abuse victimization	Yes	6	35.3	8	44.4	0.305, 0.418
	No	11	64.7	10	55.6	

Note: χ^2^ = Chi-square analyses.

**Table 3:** Observed means and standard deviations for outcome measures at pre, post, and follow-up tests.

Measures		Pre-Test		Post-Test		1st-Month Follow-Up		3rd-Month Follow-Up		6th-Month Follow-Up	
	Group	M	SD	M	SD	M	SD	M	SD	M	SD
IES-R											
Intrusion	IG	16.47	7.50	4.17	3.02	4.11	2.84	3.35	2.23	3.17	1.62
	CG	20.05	6.24	17.55	4.91	14.94	4.47	8.44	5.89	8.22	5.42
Avoidance	IG	17.17	4.65	10.41	5.43	7.76	4.75	9.23	4.58	9.17	5.05
	CG	17.83	4.01	17.44	2.52	17.16	3.05	11.72	4.36	10.5	5.89
Hyperarousal	IG	12.29	5.14	3.11	3.37	2.58	1.93	2.11	1.99	1.88	1.90
	CG	13.72	4.38	12.00	3.08	10.72	2.86	6.00	4.74	4.83	3.91
Total	IG	45.94	14.41	17.7	9.53	14.47	6.50	14.7	6.14	14.23	6.66
	CG	51.61	10.97	47.00	8.68	42.83	7.97	26.16	13.20	23.55	13.47
PTGI	Group	M	SD	M	SD	M	SD	M	SD	M	SD
Change in self-perception	IG	15.00	3.92	45.11	3.78	42.41	4.33	42.64	4.18	41.35	5.23
	CG	14.94	3.60	14.44	3.82	15.66	5.41	9.33	4.08	12.05	3.38
Change in life philosophy	IG	11.76	2.48	27.64	2.49	24.05	5.52	25.35	4.22	24.88	3.40
	CG	8.16	2.89	7.05	3.03	8.94	3.60	7.55	3.01	6.66	2.37
Change in relationships	IG	6.29	1.82	20.76	2.92	19.17	3.32	18.82	3.28	19.47	3.67
	CG	6.33	2.80	4.83	2.50	5.05	3.20	4.11	2.67	5.00	1.78
Total	IG	33.05	5.35	93.52	4.61	85.64	11.23	86.82	10.54	85.70	11.40
	CG	29.44	6.84	26.33	7.49	29.66	8.85	21.00	8.87	23.72	5.72

Note. IG: Intervention group; CG: Control group; M = Means of total IES-R and PTGI and their subscale scores; SD = Standard deviation.

**Table 4:** Repeated Measures ANOVA for IES-R and PTGI Scores (Pre-, Post-, 1st-, 3rd-, and 6th-Month Follow-Up) and Effect Sizes.

Measures	F_Time	F_Group	F_Group × Time	p_Time	p_Group	p_Group × Time	ηp^2^
IES-R–Intrusion	54.297	52.453	9.445	<0.001	<0.001	<0.001	0.622
IES-R–Avoidance	22.175	17.751	8.660	<0.001	<0.001	<0.001	0.402
IES-R–Hyper-arousal	57.547	41.562	10.611	<0.001	<0.001	<0.001	0.636
IES-R–Total	68.742	53.725	14.901	<0.001	<0.001	<0.001	0.676
PTGI–Change in self-perception	105.900	603.085	136.877	<0.001	<0.001	<0.001	0.762
PTGI–Change in life philosophy	35.078	431.625	44.170	<0.001	<0.001	<0.001	0.515
PTGI–Change in relationships	50.531	287.525	79.879	<0.001	<0.001	<0.001	0.605
PTGI–Total	112.361	607.320	150.801	<0.001	<0.001	<0.001	0.773

Note. F_time = Main effect of time; F_group = Main effect of group; F_group × time = Interaction effect between group and time; p = significance level; ηp^2^ = partial eta-squared.

## Data Availability

The datasets presented in this article are not readily available due to ethical and privacy restrictions, as the data contain sensitive information related to intimate partner abuse and mental health. Requests to access the datasets should be directed to the corresponding author upon reasonable request.
